# A Bayesian generating function approach to adverse drug reaction screening

**DOI:** 10.1371/journal.pone.0297189

**Published:** 2024-01-19

**Authors:** Tom Northardt

**Affiliations:** FAST Labs, BAE Systems Inc., Merrimack, NH, United States of America; University of Colorado Denver Skaggs School of Pharmacy and Pharmaceutical Sciences, UNITED STATES

## Abstract

Determining causality of an adverse drug reaction (ADR) requires a multifactor assessment. The classic Naranjo algorithm is still the dominant assessment tool used to determine causality. But, in spite of its effectiveness, the Naranjo algorithm is manually intensive and impractical for assessing very many ADRs and drug combinations. Thus, over the years, many “automated” algorithms have been developed in an attempt to determine causality. By-and-large, these algorithms are either regression-based or Bayesian. In general, the automatic algorithms have several major drawbacks that preclude fully automated causality assessment. Therefore, signal detection (or causality screening) plays a role in a “first pass” of large ADR databases to limit the number of ADR/drug combinations a skilled human further assesses. In this work a Bayesian signal detector based on analytic combinatorics is developed from a point of view commonly adopted by engineers in the field of radar and sonar signal processing. The algorithm developed herein addresses the commonly encountered issues of misreported data and unreported data. In the framework of signal processing, misreported ADRs are identified as “clutter” (unwanted data) and unreported ADRs are identified as “missed detections”. Including the aforementioned parameters provides a more complete probabilistic description of ADR data.

## 1. Introduction

A significant concern of pharmaceutical companies and government regulators is determining the cause of adverse drug reactions (i.e. causality assessment). Some level of causality assessment must be complete before any new drug can be approved by government regulators such as the Federal Drug Association (FDA). However, causality assessment is a very challenging problem due to many factors. Some challenges arise due to patients taking multiple medications currently, comorbidities, or drug-on-drug interactions. Consider a simple example where a patient is simply taking two medications where one is designed to lower blood pressure and another designed to control heart rate due to atrial fibrillation. If the patient presents with an abnormally low blood pressure, is the cause an adverse drug reaction due to one of the medications or due to the comorbidity of atrial fibrillation? Such scenarios can be compounded greatly especially in the elderly population where concurrently taking a dozen medications is common. However, the challenge remains that pharmaceutical companies and government regulators must reasonably assess causality to ensure the safe approval of medications.

Due to the challenges associated with causality assessment, determining causality of an adverse drug reaction (ADR) is still a multifactor assessment that, eventually requires and expert human in the assessment. Several older causality assessment tools are question-based [1, 2]. The classic Naranjo algorithm [2] is still the dominant assessment tool used to determine causality. It is a simple 10-question tool that numerically evaluates the likelihood of an ADR to a specific drug while considering factors such as timing, previous exposure, de-challenge, and re-challenge. These factors, especially de-challenge and re-challenge, help to isolate the effects of any one particular medication. But, in spite of its effectiveness, the Naranjo algorithm is impractical for assessing very many ADRs and drug combinations due to its highly manual nature. Thus, over the years, many “automated” algorithms have been developed in an attempt to determine causality. By-and-large, the automated algorithms are either regression-based [3–5] or Bayesian [6–9]. Regression-based approaches estimate coefficients of a mathematical model of several linear parameters. The work of [3], for example, parameterized an exponential probability density function assumed to represent ADR entries with three linear independent variables. Subsequent training was performed to estimate coefficients of the independent variables that best fit training ADR data. Alternatively, Bayesian-based algorithms generally retain the full form of any probability density functions and compute statistics of a posterior probability density.

However, in general, the automatic algorithms have several major drawbacks including poor specificity, require highly accurate estimates of ADR incidence, and can be very complex [10, 11]. Hence, the predominant mode of assessing causality is still expert judgment even though great disparity can be seen among physician assessment [12].

Given that the predominant mode of assessing causality requires a human in the loop, limiting the number of assessments performed to only those that exhibit a statistically significant likelihood of causation is advantageous. Therefore, signal detection (or screening) [6–9] can play a role in a “first pass” of large databases such as the FDA’s Adverse Event Reporting System (FAERS) to allow skilled humans to focus on only the very most likely causal relationships between drugs and ADRs. Several seminal works have shown that automatic algorithms for signal detection can scale to large sets of data and provide a great reduction in human workload [7, 13, 14] The work of [6] established a Bayesian neural network while the work of [14] used an empirical Bayesian approach. However, neither algorithm accounted explicitly for the reality that ADRs can be misreported or not reported at all. Given that ADRs are often entered by error-prone humans, incorporating such variables in a statistical model seems eminently reasonable.

In this work a Bayesian signal detector based on analytic combinatorics (BacSD) is developed from a point of view commonly adopted by engineers in the field of signal processing [15]. Many engineering applications commonly contend with the need to discern the presence of a signal amid “noise” through employment of statistical models. Some ubiquitous examples are radar and sonar applications [15, 16]. The algorithm developed herein is Bayesian in nature but leverages solutions to the frequently encountered issues of misreported data and unreported data. In the framework of signal processing, misreported ADRs are identified as “clutter” (unwanted data) and unreported ADRs are identified as “missed detections”. Additionally, the BacSD uses a compact generating function mathematical framework popular in analytic combinatorics [16]. Analytic combinatorics is the field of study concerned with statistical solutions to combinatoric problems where many combinations of events are common. ADR screening is a natural application as there are many possible combinations of ADRs, drugs, and counting of such. Analytic combinatorics frequently uses probability generating functions which are the z-transform of a probability mass function. Handling independent statistical variables and compactly representing probabilistic information with probability generating functions is quite convenient. Since screening ADRs in large tables in largely a counting problem, analytic combinatorics, is highly applicable.

The chief offerings of BacSD are the following. First, it accounts for misreported ADRs through a Poisson clutter model and unreported ADRs through a probability of detection (*p*_*d*_) parameter. This feature is different from the prior seminal works of [7, 13, 14]. This allows one to estimate the true number of ADRs for a particular drug/reaction combination and helps to decrease the number of highly ranking ADR/drug pairs. Second, BacSD must only estimate a single parameter from the data whereas previous work [14] must perform a much higher dimensional search for parameter fitting. Third, it exhibits a steep ranking drop-off which makes deciding when ADR numbers are uninteresting much easier whereas previous work tends to have a much more gradual monotonic descent. Lastly, it helps to characterize when ADRs are actually interestingly small. That is, some numbers of ADRs that are small will be identified as worthy of further investigation too–not simply the large numbers which are obvious. Including the aforementioned parameters provides a more complete probabilistic description of ADR data accounting for the reality that not all ADRs are reported and, those that are, may not be reported correctly.

This manuscript is organized in the following way. Section 2 presents the mathematical approach used to develop BacSD. Section 2 will describe causality screening of ADRs with a signal processing analogy. Modelling assumptions will be specified and justified. The generating functions that form the core of the algorithm will be presented as well as the methodology for estimating the sole algorithm parameter; the clutter mean. Section 3 will share some results using both simulated data and data obtained from the Side Effect Resource database (SIDER). The SIDER database was used for evaluation due to its significantly lower level of interface complexity. Section 4 will include a discussion on BacSD’s modeling flexibility, computational complexity, and computational overflow. Finally, Section 5 provides conclusions and future work.

## 2. Methods

The two ultimate objectives of this section are 1) to derive a Bayesian posterior probability generating function (PGF) for the number of ADRs for each ADR/drug pair given the observed number of ADRs, and 2) to statistically compare the estimate of the true number of ADRs to the distribution of the misreported ADRs (the clutter) for ranking.

### 2.1 ADR detection analogy to signals in noise

From an engineering signal processing point of view, there are generally three data quantities contributing to an observation model; the signal of interest, noise, and clutter. The signal of interest is the signal one wishes to measure or receive. Noise is generally random corruptive data contributions and perturbations induced by the measurement system. Clutter, however, is usually one or many legitimate signals that are often conflated with the sole signal of interest. This work assumes that when adverse drug reactions (ADRs) are reported correctly they are not generally somehow subsequently corrupted and, thus, noise is excluded from the model herein. Said another way, if an ADR is correctly reported, one does not expect that somewhere down the reporting line that it will become erroneous. The signal of interest is the ADR count while clutter are all ADRs that are erroneously reported due to human error in the reporting process. Thus, the following mathematical model describes any ADR/drug pair

$$m_{ij}=p_{d}n_{ij}+c_{ij}$$ (1)

where *m*_*ij*_ is the observed count (inclusive of erroneous reports) for the *i*^*th*^ ADR and *j*^*th*^ drug pair, *n*_*ij*_ is the true count for the *i*^*th*^ ADR and *j*^*th*^ drug pair, *p*_*d*_ is the probability of detection (a numerical value between 0 and 1) and *c*_*ij*_ is superposed clutter representing erroneous reports. The true count is unknown due to misreporting and non-reporting.

### 2.2 Modelling assumptions and justifications

This work imposes a uniform prevalence of erroneous ADR reporting among ADR/drug pairs. That is, this work hypothesizes that human entry error of ADRs does not favor some ADRs over others. Thus, all clutter values of *c*_*ij*_ are assumed to be statistically independent, identically distributed, and independent of *n*_*ij*_, and drawn from a common Poisson distribution with unknown mean 03b *λ*_*c*_. Poisson distributions are commonly used distributions for modelling counts [13, 14] and *λ*_*c*_ is estimated from the data. Similarly, *n*_*ij*_ is also assumed to be drawn from a Poisson distribution. Since one *does* expect significant dissimilarity between ADR/drug pairs due to causal relationships, the Poisson mean of *n*_*ij*_ is assumed to be unknown *λ*_*ij*_ but specific to its cell and with known prior distribution, *p*(*λ*_*ij*_). The use of a known prior distribution *p*(*λ*_*ij*_) reflects that one can likely make an educated guess for a range of values of *λ*_*ij*_ given a specific ADR/drug count, *m*_*ij*_. For example, given an ADR count of *m*_*ij*_ = 100 for a given ADR/drug pair, one might expect that the mean *λ*_*ij*_ is closer to 100 than, say, 1. In the work herein, a Gaussian prior is assumed, $\lambda_{ij}∼N({\bar{\lambda }}_{ij},\sigma_{\lambda }^{2})$, with a mean, ${\bar{\lambda }}_{ij}=m_{ij}$ equal to the observed ADR count and a variance, $\sigma_{\lambda }^{2}$ chosen to contain 50% of the interval [1,*m*_*ij*_] within two standard deviations. However, a uniform prior could be used in the absence of any other information.

### 2.3 The probability generating function model

The objective in this section will be to arrive at a Bayesian posterior probability generating function (PGF) for *N* =*n*_*ij*_, conditioned on *M* = *m*_*ij*_ observations that includes clutter modelling and a probability of detection. This PGF can be used to estimate the true number of adverse drug reactions (ADRs), ${\hat{n}}_{ij}$. With an estimate of the true number of ADRs in hand, it can be statistically compared to the misreported ADRs (the clutter) and ranked according to its likelihood of originating from the distribution of misreported ADRs.

The analytic combinatorics PGF model used to develop BacSD was adapted from [16] where it was originally introduced in the context of tracking radar and sonar objects. PGFs encode probabilistic information and are, essentially, Z-transforms of probability mass functions (PMFs). Exact probabilities are recovered by taking successive derivatives (or cross derivatives for multiple variables) of the PGFs and evaluating them at zero [16]. There are several advantages to using PGFs. First, they offer a compact mathematical formulation. Second, they support functional composition when scaling from a single realization of a random variable to, say, *m*_*ij*_ independent realizations. And lastly, they support superposed models (i.e., sums of independent random variables) very easily through a product of PGFs. The salient modelling components are introduced in this section but a more complete pedagogy can be found in the reference [16].

The four random variables being modelled within BacSD are shown below in Table 1.

**Table 1:** Modelled statistical variables within BacSD. *C* is the unknown number of erroneously reported ADRs (clutter). *M* is the observed number of ADRs for any given ADR/drug pair. *N* is the unknown number of true ADRs. *λ* is the unknown mean of the distribution from which the true ADRs were drawn.

Random Variable	Description
*C*	Number of Poisson misreported ADRs
*M*	The observed number of Poisson ADRs
*N*	The true number of Poisson ADRs
*λ*	Unknown Gaussian mean of the true number of ADRs

True ADR reports and misreported ADR reports are assumed to be statistically independent. That is, one assumes there is no logical or causal relationship between the two processes that generate them. Furthermore, individual ADR reports are assumed statistically independent from each other. That is, one assumes that one ADR report does not causally affect the next. In reality, ADRs do have an interrelationship. For example, an ADR of low blood pressure can result in an ADR of light-headedness. This interrelationship can be modelled as well but is omitted for the first development of BacSD. Given the assumptions above, the generating function that probabilistically describes the entire model will be a product of two individual generating functions; one for the true ADR reporting process and one for the misreported (clutter) ADR process (*G*_*MNλC*_ = *G*_*MNλ*_*G*_*C*_). In this section, the derivation begins with the PGF for a single ADR observation with specified probability of detection and builds upward using the mechanics of generating functions to the full Bayesian posterior PGF of all considered variables.

For now, consider just a single ADR/drug pair. The PGF for a single observation with specified probability of detection, *p*_*d*_ is

$$G_{M|N}(w)=\sum_{m=0}^{1}\mathrm{Pr}\{M=m|N=1\}w^{m}=1-p_{d}+p_{d}w$$ (2)

where *w* is the indeterminant variable (i.e. integration or dummy variable), *p*_*d*_ is the probability of detection, *M* is the number of observed ADRs, and *N* is the number of true ADRs. The values of *N* and *M* can take on any integer value from 0 to infinity. However, the probability of detection parameter, *p*_*d*_, is strictly limited to the domain [0,1]. A 0 probability of detection implies no ADRs are reported. A probability detection value of 1 implies all ADRs are reported. Reality is somewhere in between.

The encoded probabilistic information in Eq (2) by the PGF is the probability of *not* reporting the ADR given an ADR occurred (*i*.*e*., Pr{*M* = 0|*N* = 1}). That probability is 1−*p*_*d*_ and the probability of reporting the ADR given an ADR occurred (*i*.*e*., Pr{*M* = 1|*N* = 1}) is *p*_*d*_. This captures the reality that not all ADRs will be reported. Extending to multiple independent ADR realizations, *N*>1, *M*>1 (*i*.*e*., Pr{*M* = *m*|*N* = *n*}) is accomplished simply through the *n*^*th*^ product *G*_*M*|*N*_(*w*)^*n*^ for *n*>1. This fact will be employed shortly.

The primary tactic taken herein is to first develop a joint PGF for the clutter mean, number of true ADRs, and number of observed ADRs. This will encode all the joint probabilistic information. Once developed, it can be subsequently reduced to a marginal PGF of just the true number of ADRs conditioned on those observed. This final marginalized PGF is used to provide probabilistic estimates of the true number of ADRs.

To develop the joint PGF of *λ*,*N*,*M* consider the following PMF that represents the joint probabilistic mass function for the random variable of observed ADRs, *M*, the random true number of ADRs, *N*, and random unknown true ADR mean, *λ*:

$$\mathrm{Pr}(\lambda ,N,M)=\mathrm{Pr}\{N=n_{ij},M=m_{ij},\lambda =l_{ij}\}$$ (3)

Through the PGF mechanics of analytic combinatorics, the Bayesian probability generating function of *N* conditioned on the observation *m*_*ij*_ will be developed. The probability generating function (PGF) for the three aforementioned variables is,

$$G_{\lambda NM}(s,z,w)=\sum_{l=0}^{L_{\max}}\sum_{n=0}^{N_{\max}}\sum_{m=0}^{M_{\max}}\mathrm{Pr}\{N=n,M=m,\lambda =l\}s^{l}z^{n}w^{m}$$ (4)

where *s*,*z*,*w* are the indeterminant variables used in the transform, *L*_max_, *N*_max_, *M*_max_ are specified, and the (*i*,*j*) subscripts have been dropped for simplicity. One can see that to uncover any individual probability, *N* = *n*, *M* = *m*, *λ* = 1, one simply computes the mixed derivative ${\left(l!\right)}^{-1}{\left(n!\right)}^{-1}{\left(m!\right)}^{-1}{\left[\partial^{l+n+m}G/\partial^{l}s\partial^{n}z\partial^{m}w\right]}_{s=z=w=0}$ scaled by ${\left(l!\right)}^{-1}{\left(n!\right)}^{-1}{\left(m!\right)}^{-1}$ and evaluates the result at (0,0,0).

Several more steps are required to obtain the PGF for *N* conditioned on *M* and *λ*. First,

$$G_{\lambda NM}(s,z,w)=\sum_{l=0}^{L_{\max}}\sum_{n=0}^{N_{\max}}\sum_{m=0}^{M_{\max}}\mathrm{Pr}\{M=m|N=n,\lambda =l\}\mathrm{Pr}\{N=n,\lambda =l\}s^{l}z^{n}w^{m}$$ (5)

$$=\sum_{l=0}^{L_{\max}}\sum_{n=0}^{N_{\max}}\sum_{m=0}^{M_{\max}}\mathrm{Pr}\{M=m|N=n\}\mathrm{Pr}\{\lambda =l\}\mathrm{Pr}\{N=n|\lambda =l\}s^{l}z^{n}w^{m}$$ (6)

$$=\sum_{l=0}^{L_{\max}}\sum_{n=0}^{N_{\max}}\sum_{m=0}^{M_{\max}}\mathrm{Pr}\{M=m|N=n\}w^{m}\mathrm{Pr}\{\lambda =l\}\mathrm{Pr}\{N=n|\lambda =l\}s^{l}z^{n}$$ (7)

Given that the case of Pr{*M* = *m*|*N* = *n*} was discussed earlier as *n* products of Pr{*M* = (0,1)|*N* = 1}, the last equation above can be written as,

$$=\sum_{l=0}^{L_{\max}}\sum_{n=0}^{N_{\max}}{\left(\sum_{m=0}^{1}\mathrm{Pr}\{M=m|N=1\}w^{m}\right)}^{n}\mathrm{Pr}\{N=n|\lambda =l\}z^{n}\mathrm{Pr}\{\lambda =l\}s^{l}$$ (8)

Moving the *z*^*n*^ term inward allows grouping with the innermost probabilities and reveals a composition of generating functions,

$$=\sum_{l=0}^{L_{\max}}\sum_{n=0}^{N_{\max}}\mathrm{Pr}\{N=n|\lambda =l\}{\left(zG_{M|N=1}(w)\right)}^{n}\mathrm{Pr}\{\lambda =l\}s^{l}.$$ (9)

Again, since each ADR report is assumed independent of any other, another composition of generating functions occurs resulting in,

$$=\sum_{l=0}^{L_{\max}}\mathrm{Pr}\{\lambda =l\}G_{N|\lambda }(zG_{M|N=1}(w))s^{l}.$$ (10)

If the true number of ADRs is Poisson distributed with mean *λ* = *l*, *G*_*N*|*λ*_ will have the form

$$G_{N|\lambda }(z)=e^{-l+lz}.$$ (11)

And, if misreported ADRs are contributing to the observations as Poisson distributed clutter, *G*_*c*_(*w*) will have a similar form

$$G_{c}(w)=e^{-\lambda_{c}+\lambda_{c}w}.$$ (12)

Given that the true number of ADRs is considered independent of the misreported ADRs, *G*_*N*|*λ*_(*z*) can be replaced by itself multiplied by *G*_*c*_(*w*) to yield

$$G_{\lambda NM}(s,z,w)=\sum_{l=0}^{L_{\max}}\mathrm{Pr}\{\lambda =l\}e^{-\lambda_{c}+\lambda_{c}w-l+lz(1-p_{d}+p_{d}w)}s^{l}.$$ (13)

The above is the joint PGF for *λ*,*N*,*M*. The next step is to develop the joint Bayesian PGF for *N* and *λ* conditioned on a specific observation *M* = *m*.

According to [16], the joint Bayesian PGF for *N* and *λ* is obtained by

$$G_{\lambda N|M=m}(s,z)=\frac{[w^{m}]G_{\lambda NM}(s,z,w){|}_{w=0}}{[w^{m}]G_{\lambda NM}(1,1,w){|}_{w=0}}$$ (14)

where [*w*^*m*^] is an abbreviated notation for the *m*^*th*^ derivative with respect to *w* scaled by 1/*m*!. Evaluation of the denominator at (1,1,0) integrates out all random variables except for *w*. Using the above,

$$G_{\lambda N|M=m}(s,z)=\frac{\sum_{l=0}^{L_{\max}}\mathrm{Pr}\{\lambda =l\}{\left(lzp_{d}+\lambda_{c}\right)}^{m}e^{-\lambda_{c}+l+lz(1-p_{d})}s^{l}}{\sum_{l=0}^{L_{\max}}\mathrm{Pr}\{\lambda =l\}{\left(lp_{d}+\lambda_{c}\right)}^{m}e^{-\lambda_{c}+l+l(1-p_{d})}}$$ (15)

where the denominator term evaluates to a constant function of *m* and is simply a scaling factor that will be represented as *θ*(*m*).Finally, the PGF of *N* alone is obtained by evaluating the above at *s* = 1 to integrate over *λ*,

$$G_{N|M=m}(z)=\theta (m)\sum_{l=0}^{L_{\max}}\mathrm{Pr}\{\lambda =l\}{\left(lzp_{d}+\lambda_{c}\right)}^{m}e^{-\lambda_{c}+l+lz(1-p)}.$$ (16)

Therefore, given an observed number of ADRs, *m*, the PMF for *N* is obtained by

$$\mathrm{Pr}\{N=n|M=m\}={\left[z\right]}^{m}G_{N|M=m}(z){|}_{z=0}$$ (17)

Eq (17) compactly models misreported ADRs through clutter mean *λ*_*c*_ and unreported ADRs through probability of detection *p*_*d*_. From the above, an estimate of *N* can be obtained several ways. One way would be to take the mean of the PMF of *N*. This would be known as the Minimum Mean Squared Error (MMSE) estimate (Van Trees, 2013). Another way would be to take the mode of the PMF of *N*. This would be known as the Maximum A Posteriori (MAP) estimate (Van Trees, 2013). In this work, the MAP estimate is used. MMSE estimates are generally poor when the PMF is multimodal. However, multimodality of the PMF was not verified in this work and only unimodal PMFs were encountered. Thus, an MMSE estimate may perform equally well.

As mentioned previously, the probabilities Pr{*λ* = *l*} are assumed to be from a Gaussian distribution with known mean and chosen variance, $\lambda_{ij}∼N({\bar{\lambda }}_{ij},\sigma_{\lambda }^{2})$, with a mean, ${\bar{\lambda }}_{ij}=m_{ij}$ equal to the observed ADR count and a variance, $\sigma_{\lambda }^{2}$ chosen to contain 50% of the interval [1,*m*_*ij*_] within two standard deviations. All of this information, along with an observed number of ADRs, completely specifies *G*_*N*|*M* = *m*_(*z*). Given an estimate of $N,{\hat{n}}_{ij}$, and an estimate of the mean of the Poisson misreported ADR distribution, ${\hat{\lambda }}_{c}$, a log() ranking score for the statistical likelihood of the estimated true ADRs is

$$\alpha (m_{ij})=\log(1-\mathrm{Pr}\{N={\hat{n}}_{ij};{\hat{\lambda }}_{c}\})$$ (18)

where the distribution used to compute $\mathrm{Pr}\{N={\hat{n}}_{ij};{\hat{\lambda }}_{c}\}$ is the Poisson distribution with mean ${\hat{\lambda }}_{c}$. The log() taken above is similar to the metric used in [14]. Eq (18) above will produce larger values for *N* values with low probabilities of originating from the misreported (clutter) ADR distribution. Thus, large values of *N* will rank highly but, depending on the value of ${\hat{\lambda }}_{c}$, even small values of *N* may produce larger values *α*(*m*_*ij*_). For example, if ${\hat{\lambda }}_{c}=10$, a value for *N* = 1 would have approximately the same probability of occurring from the misreported ADR distribution as *N* = 19.

The next Subsection discusses how BacSD estimates ${\hat{\lambda }}_{c}$ from a table of ADR/drug pair values.

### 2.4 Estimating the clutter mean (*λ*_*c*_)

The misreported ADRs that are modelled as clutter are assumed to be independent observations from a common Poisson distribution with an unknown mean, *λ*_*c*_. In order to assess the probability of the true number of any ADR/drug pair, ${\hat{n}}_{ij}$, an estimate of *λ*_*c*_ must be obtained. The approach for obtaining such an estimate can be one of many. The approach taken herein is similar in spirit to [14] but common to signal processing approaches [15].

**Fig 1:**
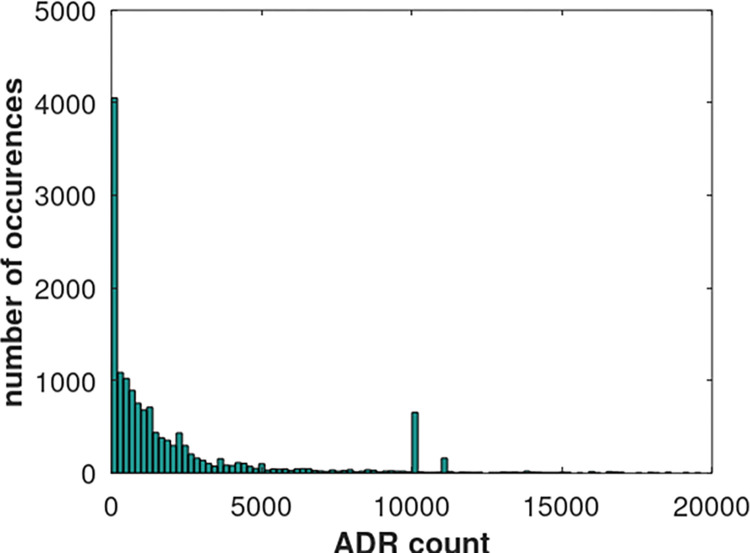
Sample data set from the SIDER database containing over 30 million records. Very few ADRs have a significant number of reports. Approximately 30% of the ADR counts are less than 200.

From the additive point of view that the number of ADR observations is the sum of the true number of ADR observations plus misreported observations, one expects that ADR values will be predominantly small with a few large values. The small values originate from the misreported ADRs that is assumed to be a common process among all reports. The large values originate from the significance of causality that BacSD and other algorithms are attempting to screen for. Fig 1 below shows a histogram of sample SIDER data generated by the current author and used in the results section to analyse BacSD. The figure below shows a histogram of ADR counts from approximately 30 million records from the SIDER database. One can clearly see the preponderance of ADR counts that are less than 2500. In fact, almost 30% are less than 200. There are very, very few large values. This distribution helps to justify the assumptions mentioned earlier.

The approach taken herein is to systematically remove the “signal” contributions until the remaining ADR reports yield a high probability of originating from a Poisson distribution with mean, ${\hat{\lambda }}_{c}$. The value of ${\hat{\lambda }}_{c}$ that yields the highest probability of generating the clutter-only ADRs will be the final estimate of *λ*_*c*_ that is used within BacSD for assessing the probabilistic support of each ADR/drug pair’s estimate of true ADRs, ${\hat{n}}_{ij}$. The approach used herein is exactly a Maximum Likelihood approach (ML) [15]. A one-dimensional search is required over a discretized set of values for *λ*_*c*_. Such a search is not computationally burdensome and much higher dimensional searches are needed in other approaches [14]. The algorithm for estimating *λ*_*c*_ is shown below in Table 2.

**Table 2:** The Maximum Likelihood (ML) algorithm for estimating the clutter mean, *λ*_*c*_. The algorithm iteratively removes 10% of the highest count values and assesses the likelihood that the remaining originated from the clutter distribution. It performs a single dimensional search on a grid of hypothesized values of *λ*_*c*_. The vector $m=[m_{1,1},m_{1,2}\ldots m_{1,J},m_{2,1},\ldots m_{I,J}]$ is abbreviated notation to show evaluation at all values of *m*_*i*,*j*_.

1. Discretize grid for estimate of *λ*_*c*_
2. Initialize likelihood *l*_*current*_ = *l*_min_ 3. Sort all ADR observations *m*_*ij*_,∀*i*,*j* in descending order
4. Remove 10% of the highest counts
5. Compute likelihood $l(m;{\hat{\lambda }}_{c})=\prod_{i,j=1}^{I,J}p(m_{ij};{\hat{\lambda }}_{c})$ 6. If $l(m;{\hat{\lambda }}_{c})>l_{current},l_{current}=l(m;{\hat{\lambda }}_{c})$
7. Return to 2. Stop after all values of *λ*_*c*_ are evaluated.
8. Select estimate ${\hat{\lambda }}_{c}$ associated with *l*_*current*_, the maximum likelihood

## 3. Results

This section provides several performance plots for analysis of BacSD. The first subsection makes use of entirely simulated data to explicitly control the effects of the clutter model (misreported ADRs) and probability of detection (unreported ADRs). In the simulations the mean of the clutter model is set *λ*_*c*_ = 8. Random ADRs are generated with random identifiers. For the simulated data, ADR identifiers are not of significant importance as the aim is to explore the ranking of BacSD and its behaviour as a function of probability of detection. All results were obtained by implementing BacSD using open-source Octave 7.3.0.

The second subsection examines the performance of BacSD in comparison to [14] using data from the SIDER database. As one of the older, more frequently used and referenced algorithms, the algorithm offered by [14] provides a good comparator for evaluation. The clutter mean is estimated from the same SIDER data and a probability of detection is chosen to be a nominal value of 0.9.

### 3.1 Estimate of N versus P

**Fig 2:**
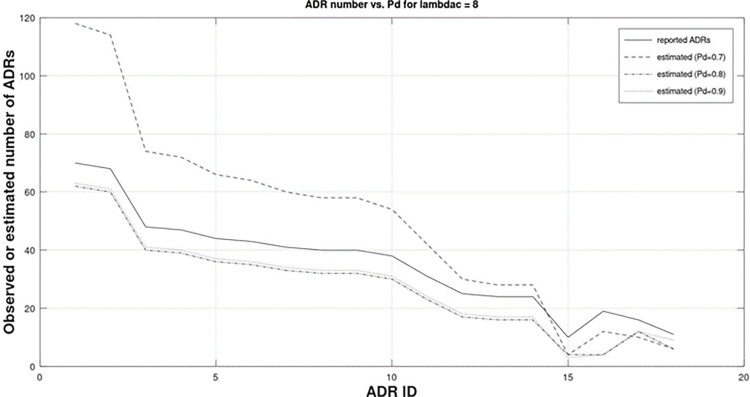
Highest 18 BacSD rankings as a function of detection probability using simulated data with a clutter mean of 8. ADRs are ranked from highest to lowest from left to right. The solid black line is the observed ADR count for each ADR shown. Non-solid lines are the estimated number of true ADRs for each ADR ID for varied detection probability. The low value at ADR ID 15 is due to ADR counts occurring having high probability of originating from clutter which allows BacSD to identify ADRs that are both “interestingly large” and “interestingly small”.

Fig 2 below can be used to examine the performance of BacSD with respect to clutter and with respect to probability of detection. The ADR IDs in Fig 2 are ordered by their BacSD ranking from highest to lowest for readability.

Recalling that the clutter model used to simulate the misreported ADRs had a mean of 8 (*λ*_*c*_ = 8), the solid black line is the number of ADR observations simulated which includes both misreported ADRs and the true number of ADRs. For example, ADR ID 5 had a total observed number of ADRs of 45. For a particular ADR ID, this means that some number of ADRs truly occurred for that particular drug/ADR pair and some did not but were erroneously reported as occurring. BacSD estimates the number of truly occurring ADRs by estimating *λ*_*c*_ and with the probability of detection, *p*_*d*_, specified. The remaining lines are the estimated number of true ADRs, ${\hat{n}}_{ij}$ (*j*≡1 for the simulation), reported for various values of *p*_*d*_. Three noteworthy behaviours can be seen which are discussed now.

First, for large numbers of reported ADRs on the left, when the probability of detection is only 0.7, the estimated number of true ADRs is quite high. For example, in Fig 2 ADR ID 5, with 45 reported ADRs, is estimated to have approximately 67 true ADRs. This is intuitively pleasing because a probability of detection of 0.7 implies that 30% of the truly occurring ADRs were not reported. Additionally, *λ*_*c*_ = 8 implies that, on average, one expects 8 misreported ADRs to contribute to the overall 45 ADRs observed. Thus, one can expect that the true number of ADRs to be greater than those observed with such a high percentage not being reported. As probability of detection increases from 0.8 and to 0.9, the estimated number of truly occurring ADRs decreases. As probability of detection increases toward 1, the estimated true number of ADRs approaches ${\hat{n}}_{ij}=m_{ij}-\lambda_{c}$.

Second, for the simulated data used, a sharp decrease in the estimated number of true ADRs can be seen for all probability of detection values near ADR ID 15. And, by its numerical order (recall the ADR IDs are ordered by their BacSD ranking highest to lowest), ADR ID 15, having a lower number of observed ADRs than those to the right of it (e.g. ADR ID 16, 17, etc…) has a higher BacSD ranking. This behaviour is different from other ADR screening tools [6–9]. Generally speaking, the ranking of “interesting” from the ADR screening tools is strictly monotonic. That is, higher observed values have higher rankings. For BacSD, this is true except for where the observed value has a high statistical likelihood of originating from the clutter (misreported ADR) distribution (in this simulation a mean of *λ*_*c*_ = 8). Thus, BacSD has as the ability to discern when the number of reported ADRs is both “interestingly large” and “interestingly small.”

Lastly, for higher values of *p*_*d*_, (greater than 0.7), estimates of the true number of ADRs are fairly close in value. This indicates that a well-known value of *p*_*d*_ is not necessary and an approximate estimate will suffice.

From an ADR analysis perspective, the abovementioned features are quite relevant. The first feature shows that BacSD produces an estimate of the number of “true” ADRs that compensates for unreported ADRs. That is, some ADRs occur but are not reported. The second feature demonstrated shows that BacSD will draw a user’s attention to ADRs that occur in both large and small number. This will allow for the possibility of identifying potentially important ADRs that do not occur frequently and would not otherwise be identified by existing algorithms such as [14].

### 3.2 SIDER data evaluation

**Fig 3:**
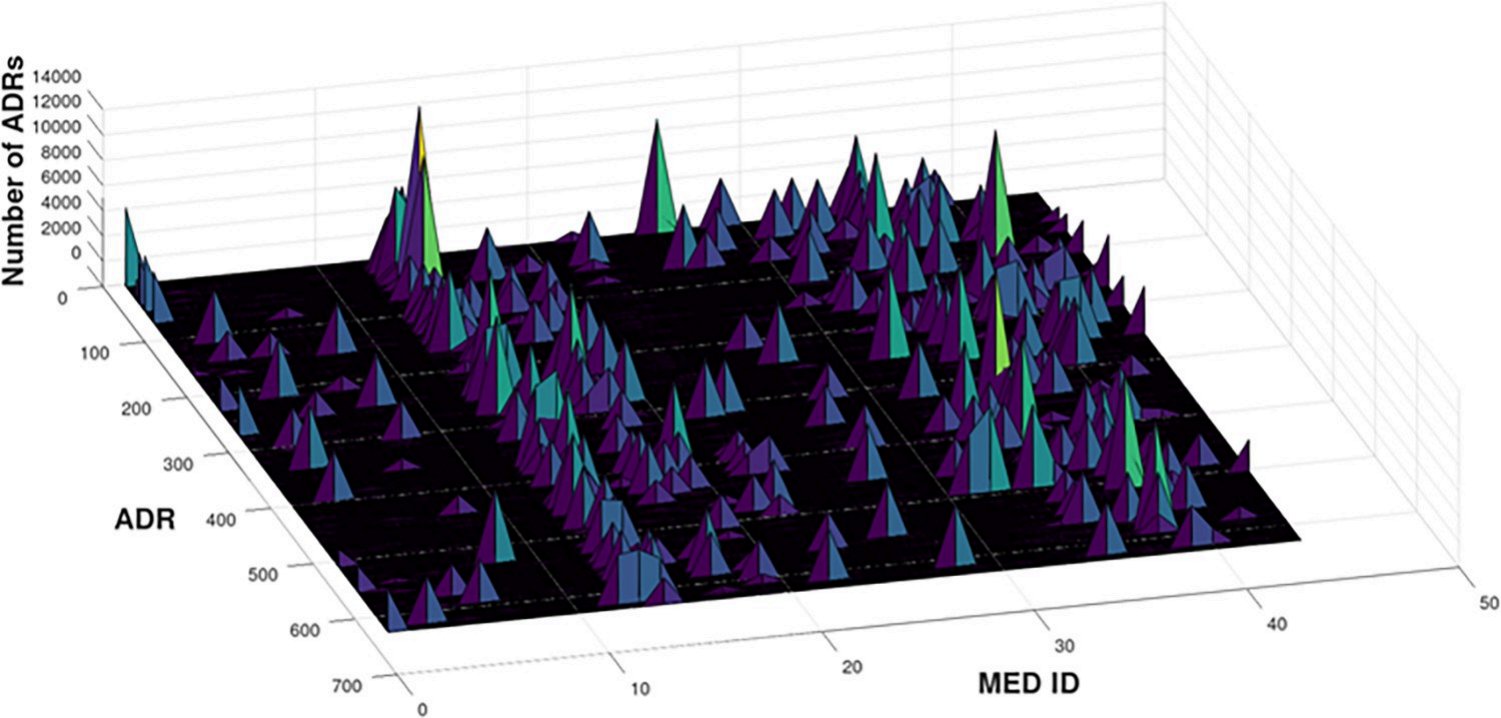
Sample MeDRA data from the SIDER database containing over 40 potential drugs (MED IDs) and over 650 potential ADRs. In this 3-dimensional view one can easily identify the relatively few high counts of ADR/drug pairs.

In this section, the performance of BacSD is compared to that of [14] (termed therein as Empirical Bayes or EB) using data from the SIDER database. For the data used, which contained reported ADRs as many as 13,000 for some drug/ADR pairs, the clutter mean was estimated to be, ${\hat{\lambda }}_{c}=43$ using the maximum likelihood method described in Section 2.4. A probability of detection was set equal to a nominal value of *p*_*d*_ = 0.9. Fig 3 below shows the number of ADRs as a function of ADR and MED ID. There are over 650 potential ADRs and over 40 potential drugs.

**Fig 4:**
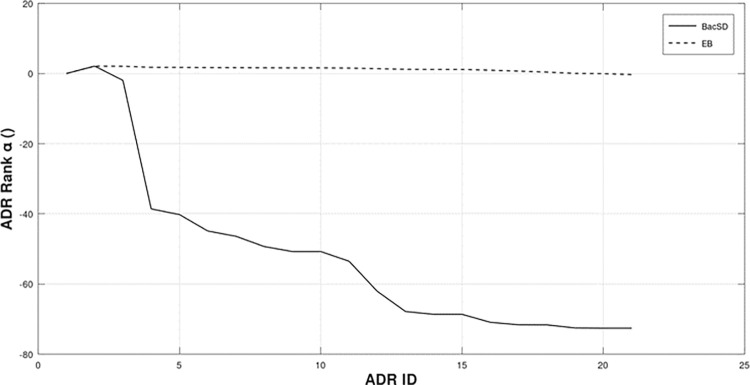
Top 15 rankings from both EB and BacSD for a probability of detection of 0.9 and estimated clutter mean of ${\hat{\lambda }}_{c}=43$. BacSD has a very steep decline of values easing the selection of a threshold between interesting and uninteresting.

Fig 4 below shows the top 15 *α*(*m*_*ij*_) ranking values of BacSD and EB ordered from highest to lowest for the 15 highest number of ADR observations. Both EB and BacSD show the same monotonic decline of rank for decreasing number of observations. However, the decline in ranking is much greater for BacSD. From a practical view, a sharper drop-off makes selecting a threshold between “interesting” and “uninteresting” much easier. For the data shown, ADR IDs with ranking values less than ADR ID 8 could arguably be excluded from further causality analysis. Making such a choice with EB is more difficult.

Additionally, a statistical threshold between “interesting” and “uninteresting” could be chosen by taking the antilogarithm of Eq (18) and classifying any ADR/drug pair as uninteresting that occurs with probability less than *p*_*h*_. Common values for *p*_*h*_ are 0.95, and 0.99.

Table 3 below lists the associated ADRs with the 15 ADR IDs shown in Fig 4. The first ADR Abdominal Pain Upper, pertains to Carnitine and is listed in the open literature as one of Carnitine’s known side effects.

**Table 3:** Top 15 ADRs ranked from EB and BacSD. Both listings are identical with the first corresponding to Carnitine. Open literature lists upper abdominal pain as a common side effect of Carnitine.

	EB	BacSD
1	Abdominal pain upper	Abdominal pain upper
2	Abdominal pain	Abdominal pain
3	Abdominal tenderness	Abdominal tenderness
4	Acute coronary syndrome	Acute coronary syndrome
5	Abdominal pain localised	Abdominal pain localised
6	Acne	Acne
7	Abdominal bloating	Abdominal bloating
8	Abdominal distension	Abdominal distension
9	Accidental injury	Accidental injury
10	Administration site reaction	Administration site reaction
11	Abdominal discomfort	Abdominal discomfort
12	Abnormal withdrawal bleeding	Abnormal withdrawal bleeding
13	Abnormal dreams	Abnormal dreams
14	Abnormal vision	Abnormal vision
15	Acute leukaemia	Acute leukaemia

From an ADR analysis perspective, Fig 4 demonstrates a very helpful performance trait of BacSD. The trait observed is a sharp decrease in ADR ranking. This makes threshold setting between ADRs that will move on to further investigation vice those that will not a much easier task. A reasonable threshold could be chosen from Fig 4 as -40. With such a threshold, only ADRs below ID 5 would be further analysed.

## 4. Discussion

### 4.1 Modelling flexibility

Compared to other ADR screening algorithms, BacSD primarily adds a clutter model to address misreported ADRs and a probability of detection to address unreported ADRs. Both of these pieces of information are used to obtain a Bayesian estimate for the true number of reported ADRs for any ADR/drug pair. This added flexibility helps to address the intuitive realities that not all ADRs will be reported correctly and not all ADRs are even ever reported. In some scenarios this will allow BacSD to identify interestingly large and interestingly small ADR values as was seen with the simulated data in Section 3.1. When a human analyst is required to further investigate causality, BacSD could help further reduce unnecessary analysis. In other scenarios BacSD may offer steeper rankings as seen in Section 3.2 to help one determine a threshold by which to discard uninteresting ADR counts.

### 4.2 Computational expense

BacSD applies a single clutter model parameter (misreported ADRs) to the entire available dataset. Other screening algorithms do similarly but the number of parameters needed to fit the distribution is often more. For example, in [14] four parameters are needed to fit the assumed underlying probability density. This requires a costly 4-dimensional search (albeit, it only needs to be done relatively infrequently). In contrast, BacSD requires just the 1-dimensional search discussed in Section 2.4. Aside from the initial model fitting, on-line computational requirements of BacSD seem to be of the same order of magnitude as other screening algorithms.

### 4.3 Computational overflow

BacSD uses multiple Poisson models which can produce very large numbers and quickly lead to computational overflow without thoughtful implementation. For example, consider the term (*lzp*_*d*_+*λ*_*c*_)^*m*^ in Eq (15). In Eq (15), *m* is the number of observed ADR counts for a particular ADR/drug pair. If the term in parenthesis is greater than one and *m* is large, say 2000, then a computational overflow is quickly encountered. One method to circumvent the overflow is to scale the ADR observation table to compress the dynamic range of the observations to within manageable computing values. However, model fitting should be completed first.

## 5. Conclusions and future work

This work presented a Bayesian analytic combinatoric adverse drug reaction (ADR) signal detector algorithm. The algorithm, named BacSD, offered two improvements over prior seminal works applied to large datasets: 1) the incorporation of missed ADR detections, and 2) the incorporation of false ADR detections.

Through the analysis of Section 4, BacSD was found to provide intuitively pleasing estimates of the true number of reported ADRs while compensating for unreported ADRs and false ADRs. BacSD also highly ranked ADRs that did not necessarily occur in large number. Thus, BacSD can help draw attention to potentially important ADRs that do not occur frequently and may not be identified by existing algorithms. Additionally, BacSD exhibited a steep drop-off in ranking value. This feature will greatly help reduce the volume of ADRs that must be further investigated by an expert human.

Given the assumptions disclosed in Section 2, the most logical direction for future BacSD work would be to incorporate compensation for drug-on-drug interaction. This could be accomplished in a few different ways. The simplest way is to modify the clutter mean *λ*_*c*_ to account for the fact that an ADR associated with a drug may have truly been the result of the drug-on-drug interaction with another drug. A more complicated approach would revisit Eq (4) to consider the joint probabilities of the true number of ADRs given observations of the multiple ADRs possibly arising from drug-on-drug interactions.

## Supporting information

S1 Appendix (DOCX)
