# Supplementary material for: A Bayesian generating function approach to adverse drug reaction screening
Source: PLoS One. 2024 Jan 19;19(1):e0297189. doi: 10.1371/journal.pone.0297189 (PMC10798640; doi:10.1371/journal.pone.0297189)
Supplement: S1 Appendix — (DOCX) [file pone.0297189.s001.docx]

# APPENDIX A

In this section, additional details for the explicit form of Equation (17) are provided under the models adopted in this work. Equation (17) is explicitly shown below for the problem modelled herein with the necessary differentiation,

$\Pr\{N=n|M=m\}|_{z=0}=\theta(m)\sum_{l=0}^{L_{\max}} \Pr\{\lambda=l\}\frac{\partial^{n}}{\partial z^{n}}\left((lzp_{d}+\lambda_{c})^{m}e^{-\lambda_{c}+l+lz(1-p_{d})}\right)|_{z=0}$. A.1

The differentiation with respect to $z$must be performed first before the entire expression is evaluated at $z=0$. Because the function to be differentiated is a product of two functions of $z$, the following differentiation chain rule is helpful

$\frac{\partial^{n}}{\partial z^{n}}=\sum_{\begin{matrix} i,j \\ i+j=n \end{matrix}}^{n} \frac{\partial^{i}f(z)}{\partial z^{i}}\frac{\partial^{j}g(z)}{\partial z^{j}}=\sum_{\begin{matrix} i=0 \\ j=n-i \end{matrix}}^{n} \frac{\partial^{i}f(z)}{\partial z^{i}}\frac{\partial^{j}g(z)}{\partial z^{j}}$. A.2

For the problem addressed herein, let $f(z)=(lzp_{d}+\lambda_{c})^{m}$and $g(z)=e^{-l-\lambda_{c}+lz(1-p_{d})}$. Given any arbitrary value of $n$(a possible value for the true ADR count) and a value of $m$(the observed ADR count), one can evaluate Equation (17) using Equation (A.1) and Equation (A.2).

# Author Details

Dr. Tom Northardt is the Technical Area Director for Maritime Systems and Technology at BAE System’s FAST Labs. Tom received his BS, MS, and Ph.D. all in Electrical Engineering from the University of Massachusetts at Dartmouth, in 2005, 2009, and 2013 respectively. His interests have been mainly focused on statistical array signal processing, multi-sensor fusion, and practical Bayesian applications. A few of his works have contributed to the medical field in the areas of breast mammography and pharmacoepidemiology.
